# Wood quality-related gene expressions of *Eucalyptus globulus *grown in a greenhouse

**DOI:** 10.1186/1753-6561-5-S7-P115

**Published:** 2011-09-13

**Authors:** Negishi Naoki, Kazuya Nanto, Kazunori Hayashi, Shinichi Onogi, Akiyoshi Kawaoka

**Affiliations:** 1 Agri-Biotechnology Research Laboratory, Nippon Paper Industries Co.Ltd

## 

Eucalyptus species constitute the most widely planted hardwood trees in temperate and subtropical regions. Their wood is used as a raw material for the production of cellulose. Eucalyptus species have fast growth rates and the ability to adapt to a broad range of geographic locations. Most importantly, Eucalyptus has been listed as one of the candidate biomass energy crops [1]. *Eucalyptus globulus* is the main hardwood species grown in pulpwood plantations in temperate regions of the world.

In this study, we focused on four kinds of key genes, 4-coumarate-CoA ligase (4CL), LIM domain transcription factor (LIM) [2], coniferaldehyde 5-hydroxylase (CAld5H) and the three catalytic units of cellulose synthase (CesA1, CesA2 and CesA3) influencing wood quality . We investigated correlation between relative expression levels of these genes and wood qualities.

We have cloned the genes encoding LIM, 4CL, CAld5H and the cellulose synthase (CesA1, CesA2 and CesA3) from *E. globulus* by the method of cDNA library screening (Fig.1). A cDNA library was constructed using mRNA purified from stems of four-month old *E. globulus* grown in the greenhouse. The expression levels of LIM in basal stems of ten independent *E. globulus* lines showed similar patterns to those of 4CL, indicating that the LIM may control 4CL expression. We investigated the correlation between gene expression levels and wood qualities such as Klason lignin (KL) content, syringyl/guaiacyl (S/G) ratio and holocellulose (HC) content.Expression of the LIM and 4CL were positively correlated with KL content. A highly significant positive correlation was observed between CAld5H expression and S/G ratio. Furthermore, a ratio of the sum of the expression levels of three CesA1, CesA2 and CesA3 to 4CL showed positive correlation with a ratio of HC/KL content that positively correlated to the chemically extracted fiber content in this woody plant. Overall, our results provide a strong foundation for manipulating candidate genes such as LIM, 4CL, CAld5H and CesA towards the production of desirable wood qualities in an extremely important biomass plant, the Eucalyptus species.
